# Time to surgery and survival in breast cancer

**DOI:** 10.1186/s12893-022-01835-1

**Published:** 2022-11-11

**Authors:** Doyeon An, Jihye Choi, Jaebin Lee, Jong-Yeup Kim, Seonguk Kwon, Jungeun Kim, Seunghee Lee, Seongwoo Jeon, Chungchun Lee, Suehyun Lee, Hyekyung Woo

**Affiliations:** 1 Department of Health Administration, Graduate School, Kongju National University, Gongju, Republic of Korea; 2 Department of Health Promotion and Behavioral Sciences, School of Public Health, University of Texas Health Science Center at Houston, Houston, USA; 3 Division of Healthcare and Artificial Intelligence, National Institute of Health, Cheongju, Republic of Korea; 4 Department of Biomedical Informatics, College of Medicine, Konyang University, Daejeon, Republic of Korea; 5 Departments of Surgery, Konyang University Hospital, Daejeon, Republic of Korea; 6 Department of Software, College of Engineering, Kongju National University, Cheonan, Republic of Korea; 7 Healthcare Data Science Center, Konyang University Hospital, Daejeon, Republic of Korea

**Keywords:** Breast cancer, Delayed treatment, Neoadjuvant chemotherapy, Surgery, Time

## Abstract

**Background:**

This study aimed to investigate the effect of the time from diagnosis to breast cancer surgery on breast cancer patients’ prognosis.

**Methods:**

Of the 1900 patients diagnosed with invasive (stage 1–3) breast cancer who underwent surgery in KUH between 2012 and 2019, 279 patients were enrolled in this study. All patients, including those who received neoadjuvant chemotherapy, were classified as Model 1 subjects, and those who received immediate surgical treatment were classified as Model 2 subjects. We conducted a Cox regression analysis to identify prognostic factors of breast cancer associated with the time from diagnosis to surgery.

**Results:**

The univariate results indicated a sharp drop in both groups’ survival rates when the time to surgery was delayed for more than 8 weeks (Model 1 *p* = 0.000; Model 2 *p* = 0.001). In the multivariate analysis, the hazard ratio (HR) of Model 1increased (HR = 6.84, 95% CI 1.06–44.25) in response to a delay in surgery of more than 8 weeks. Smoking and the American Joint Committee on Cancer (AJCC) staging system had a negative effect on breast cancer prognosis, while hormone therapy had a positive effect.

**Conclusion:**

For all patients, a delay in breast cancer surgery of more than 8 weeks was inversely associated with survival.

## Background

Interest in breast cancer prognosis continues to grow as the incidence of the disease rises [1]. Among the various prognostic factors, time from diagnosis to surgery is a key determinant of breast cancer survival and has become a central concern to patients and clinicians in recent years [2–8]. There is accumulating evidence to suggest that it affects survival outcomes in metastatic breast cancer [2, 9, 10]. Given that time to surgery is a modifiable and controllable factor contingent upon the provider’s clinical decision-making, timeliness of treatment has been proposed as a measure of quality control and assurance in the context of breast cancer treatment in addition to its clinical importance [11].

Time to surgery is particularly important for breast cancer patients. Delays in surgical treatment exacerbate patients’ anxiety [12] and contribute to adverse outcomes, such as disease progression or further postponement of adjuvant treatment [6, 8, 13, 14]. Time to surgery may vary depending on the patient’s sociodemographic and health status [13]. Efforts to reduce waiting times to surgery can enhance treatment quality and patient satisfaction [9] in addition to alleviating the patient’s psychological distress [12, 15].

Preoperative or neoadjuvant chemotherapy is used to treat patients with locally advanced breast cancer [16]. Despite its potential to downstage primary tumors that progress rapidly [17] and reduce the need for lymph-node dissection [18], patients subject to this preoperative treatment wait longer until their first surgery, which is usually recommended within three weeks of initial diagnosis [19]. Prolonged time to surgery can accelerate tumor progression and is a major threat to survival [3, 4, 7, 20]. It is therefore critical to examine the effect of patient wait times to neoadjuvant chemotherapy on breast cancer survival rates. However, few studies have compared survival rates between patients with and without neoadjuvant chemotherapy to understand the effects of the associated waiting time on breast cancer prognosis.

The present study aimed to examine the effect of the time from diagnosis to surgery on the prognosis of breast cancer [2]. The findings of this study can serve as a reference for the design of clearly defined guidelines for breast cancer care and treatment. The development of and adherence to such guidelines by oncologists will help minimize the delay in time to surgery and promote early treatment.

## Methods

### Study design

Of the 1900 patients diagnosed with stage 1–3 breast cancer who underwent surgery in KUH between 2012 and 2019, 279 were enrolled in this study. We excluded patients who had unmatched records in the cancer registration data, had not undergone surgery, or were diagnosed with stage 4 breast cancer. Patients whose American Joint Committee on Cancer (AJCC) stage was unknown were also excluded.

We confirmed through previous studies that the prognosis of patients who received immediate surgical treatment as well as patients who received neoadjuvant chemotherapy could vary depending on the interval [19, 21]. In addition, the interval between surgery may be longer for patients receiving neoadjuvant chemotherapy [16]. This study has at two perspectives. First, all patients, including those who received neoadjuvant chemotherapy, were classified as Model 1 subjects to compare the effects of the receipt and non-receipt of neoadjuvant chemotherapy on the time-to-surgery interval. Second, we analyzed patients who received immediate surgical treatment by classifying them as Model 2 subjects additionally.

### Data sources

To analyze diagnosis and treatment outcomes, we used data from the breast cancer library at Konyang University Hospital (KUH), which participated in building the Big Data Platform Network for the Korea Central Cancer Registry (KCCR). The Korea Cancer Big Data Platform is a multi-database framework constructed from electronic medical records (EMR) that include information such as diagnosis, examination results, administrative data, treatment, surgery, and national cancer registration data. We also used cancer registration data from KUH and data from the breast cancer library. Cancer registration data were used to obtain tumor characteristics and prognostic factors.

### Study variables

Surgical delays of more than 8 weeks affect the patient’s prognosis [3, 20]. Consequently, it is necessary to carefully monitor the patient status at the 8-week mark of the delay in surgery. Time to surgery is defined as the time from confirmed diagnosis to first surgery [14, 22]. The criteria for survival endpoint were implemented based on the 5-year overall survival. The intervals between diagnosis and surgery were as follows: < 2 weeks, 2–4 weeks, 4–8 weeks, and > 8 weeks.

Cancer variables in the KCCR included AJCC stage, histological differentiation, and presence of ductal carcinoma. AJCC stage is a cancer staging system used to describe the amount and spread of cancer in a patient’s body; the severity of breast cancer was classified as stage 1, 2, or 3 according to the criteria of the 8th edition of the AJCC [23]. Histological differentiation (well or moderate, poor, unknown) and presence of ductal carcinoma (yes/no) were classified according to the International Classification of Diseases, Tenth Revision Clinical Modification (ICD-10-CM) codes. Treatment variables included surgery type (mastectomy, lumpectomy), chemotherapy (yes/no), hormone therapy (yes/no), radiotherapy (yes/no), and mammogram screening (yes/no). The other variables included in the analysis were age at diagnosis (≤ or > 50 years), alcohol consumption (yes/no), smoking (yes/no), and asymptomaticity before surgery (yes/no).

### Analysis

We conducted a descriptive analysis of the distribution of patients’ demographic characteristics and tumor and treatment types by time to surgery. We used a Kaplan Meier Estimation and multivariate Cox regression analysis to investigate whether time-to-surgery intervals were related to survival and to identify predictors of time to surgery. In the multivariate analysis, we adjusted for the following variables: age at diagnosis, alcohol consumption, smoking, asymptomaticity before surgery, AJCC stage, histological differentiation, presence of ductal carcinoma, chemotherapy, hormone therapy, radiotherapy, and mammogram screening. We used R software (version 4.0.3) for all statistical analyses.

## Results

**Fig. 1:**
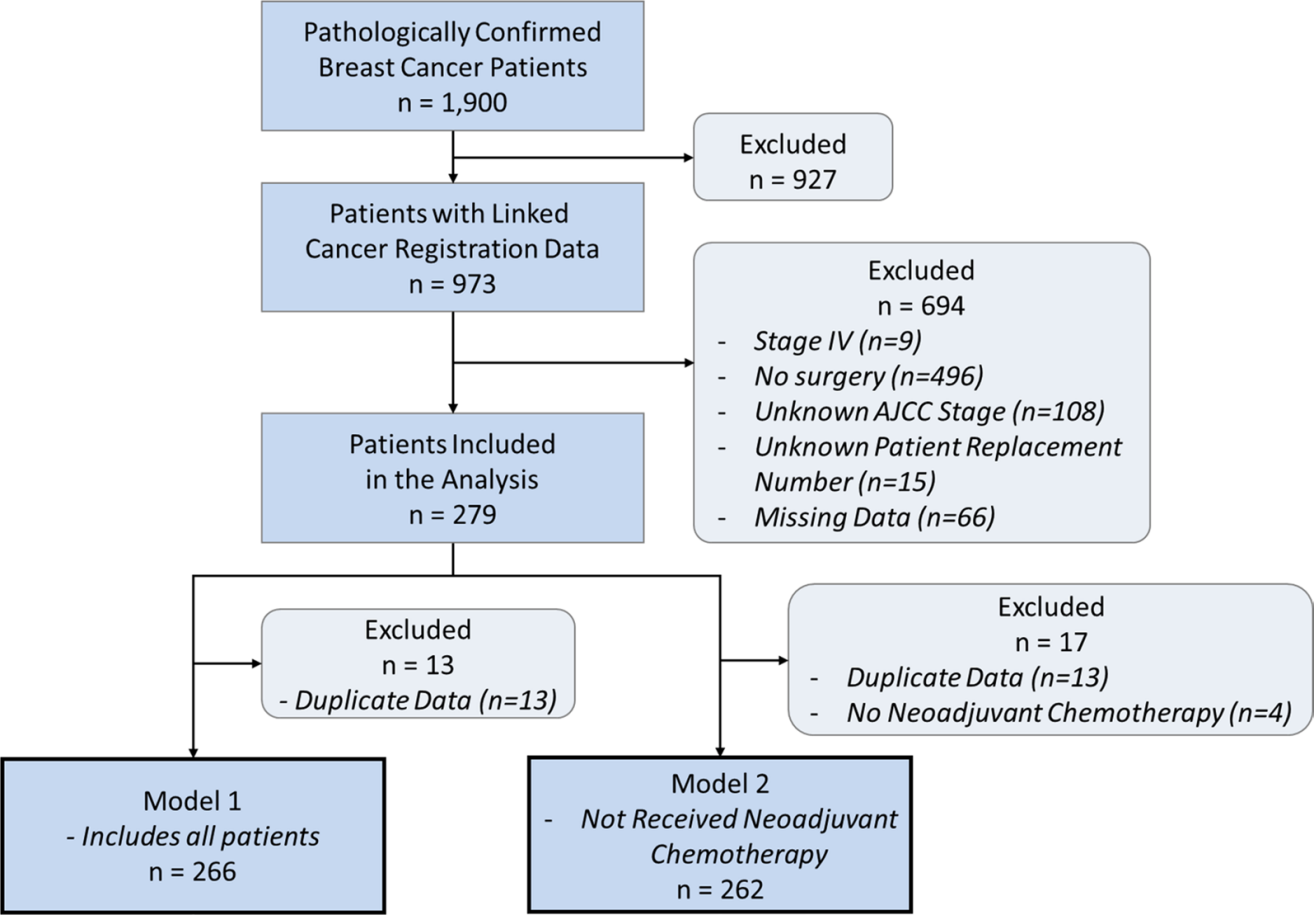
Flowchart of the study participants. *AJCC* American Joint Committee on Cancer

A total of 279 eligible patients were included in the analysis (Fig. 1). Most of the patients were aged above 50 years, and the vast majority did not smoke or consume alcohol. Prior to undergoing surgery, 33% (n = 92) of the patients waited less than 2 weeks, 42% (n = 117) waited 2–4 weeks, 16% (n = 45) waited 4–8 weeks, and 9% (n = 25) waited for more than 8 weeks.

Table 1 summarizes the demographic characteristics and tumor and treatment types for the overall patient population by time to surgery. Fewer patients were asymptomatic prior to undergoing surgery, and the number of deaths was highest in the last interval category (> 8 weeks). Stage 1 patients were the most likely to undergo surgery within 4 weeks of diagnosis. Patients with poor histological differentiation were the most likely to undergo surgery within 2 weeks. Most patients had ductal carcinoma and underwent mastectomy; the proportion of patients who received chemotherapy was lowest in the last interval category. Among those who received hormone therapy, most patients waited 2–4 weeks for surgery. Radiotherapy and mammogram screening were most common among patients who waited 2–4 weeks for surgery.

**Table 1:** Demographic, cancer, and treatment characteristics based on time to surgery n (%)

Characteristic	> 2 weeks	2–4 weeks	4–8 weeks	< 8 weeks	*p*
	(n = 92)	(n = 117)	(n = 45)	(n = 25)	
Age at diagnosis (years)					0.528
> 50	40 (35.1)	42 (36.8)	20 (17.5)	12 (10.5)	
≤ 50	52 (31.5)	75 (45.5)	25 (15.2)	13 (7.9)	
Alcohol consumption					0.387
Yes	17 (26.2)	29 (44.6)	14 (21.5)	5 (7.7)	
No	75 (35.0)	88 (41.1)	31 (14.5)	20 (9.3)	
Smoker					0.355
Yes	13 (44.8)	12 (41.4)	2 (6.9)	2 (6.9)	
No	79 (31.6)	105 (42.0)	43 (17.2)	23 (9.2)	
Asymptomatic before surgery					0.105
Yes	59 (38.8)	55 (36.2)	24 (15.8)	14 (9.2)	
No	33 (26.0)	62 (48.8)	21 (16.5)	11 (8.7)	
Death					0.002**
Yes	4 (30.8)	3 (23.1)	1 (7.7)	5 (38.5)	
No	88 (33.1)	114 (42.9)	44 (16.5)	20 (7.5)	
AJCC stage					0.000***
I	36 (35.0)	50 (48.5)	10 (9.7)	7 (6.8)	
II	46 (33.8)	48 (35.3)	33 (24.3)	9 (6.6)	
III	10 (25.0)	19 (47.5)	2 (5.0)	9 (22.5)	
Histological differentiation					0.004**
Well/Moderate	25 (31.2)	37 (46.3)	14 (17.5)	4 (5.0)	
Poor	47 (46.1)	37 (36.3)	11 (10.8)	7 (6.9)	
Unknown	20 (20.6)	43 (44.3)	20 (20.6)	14 (14.4)	
Ductal carcinoma					0.447
Yes	81 (32.9)	100 (40.7)	41 (16.7)	24 (9.8)	
No	11 (33.3)	17 (51.5)	4 (12.1)	1 (3.0)	
Surgery type					0.817
Mastectomy	80 (34.0)	96 (40.9)	38 (16.2)	21 (8.9)	
Lumpectomy	12 (27.3)	21 (47.7)	7 (15.9)	4 (9.1)	
Chemotherapy					0.581
Yes	42 (37.2)	43 (38.1)	17 (15.0)	11 (9.7)	
No	50 (30.1)	74 (44.6)	28 (16.9)	14 (8.4)	
Hormone therapy					0.010**
Yes	65 (30.5)	98 (46.0)	36 (16.9)	14 (6.6)	
No	27 (40.9)	19 (28.8)	9 (13.6)	11 (16.7)	
Radiotherapy					0.146
Yes	61 (33.3)	78 (42.6)	24 (13.1)	20 (10.9)	
No	31 (32.3)	39 (40.6)	21 (21.9)	5 (5.2)	
Mammogram					0.001***
Yes	7 (14.0)	21 (42.0)	15 (30.0)	7 (14.0)	
No	85 (37.1)	96 (41.9)	30 (13.1)	18 (7.9)	

*AJCC* American Joint Committee on Cancer

*p < 0.05, **p < 0.01, ***p < 0.001

**Fig. 2:**
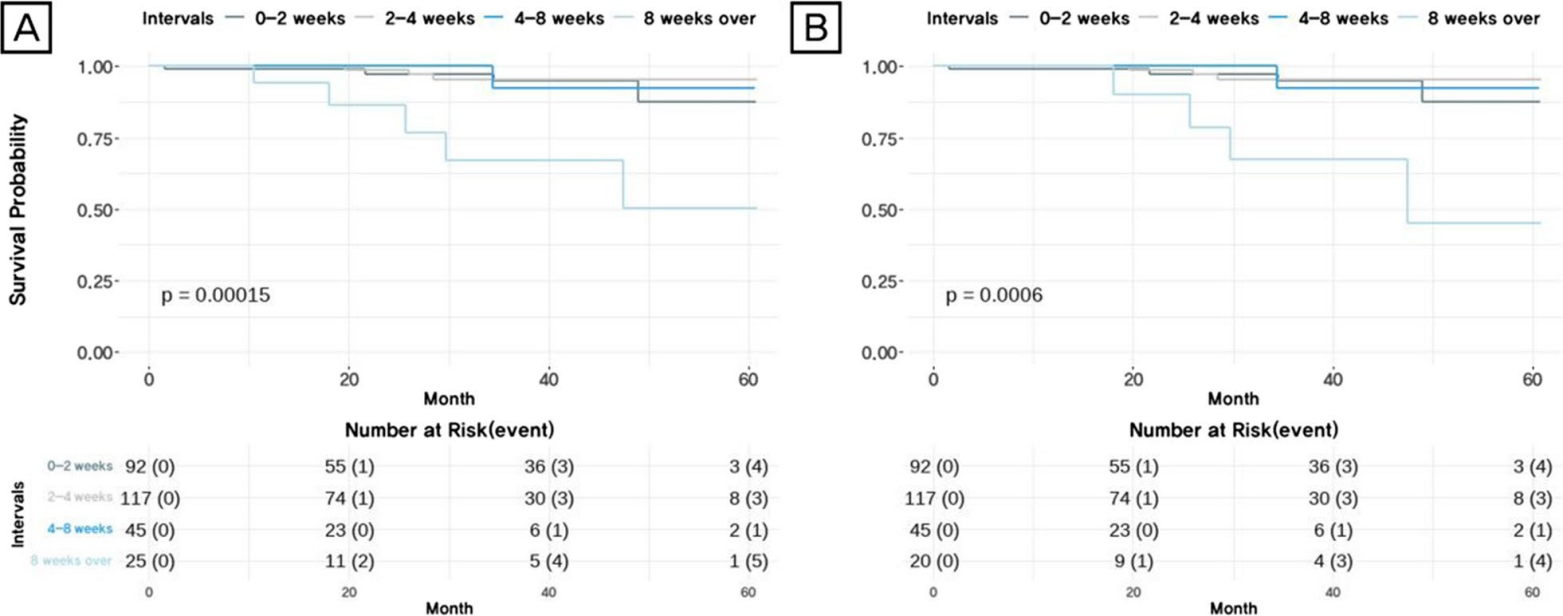
Unadjusted 5-year overall survival data. **A** All patients who underwent surgical treatment including neoadjuvant patients. **B** Patients who have not received neoadjuvant chemotherapy and have undergone surgical treatment

During the study period, a total of 13 deaths occurred in the entire cohort (A) and the group of patients who did not receive neoadjuvant chemotherapy (B), before adjusting for survival and time to surgery (Fig. 1). According to the survival analysis, a time interval of longer than 8 weeks was a statistically significant risk factor for survival (*p* = 0.00015) based on the Kaplan–Meier plot (Fig. 2A). For patients who did not receive neoadjuvant chemotherapy (B), the survival rate dropped sharply when the interval was more than 8 weeks (*p* = 0.0006) (Fig. 2B).

Table 2 illustrates the results of multivariate analysis of the effects of time to surgery on breast cancer prognosis. In Model 1, surgery delayed for more than 8 weeks was significantly associated with increased mortality (hazard ratio [HR] = 6.84, 95% confidence interval [CI] 1.06–44.25). Surgeries delayed for 2–4 weeks (HR = 1.31, 95% CI 0.21–8.22) and 4–8 weeks (HR = 1.47, 95% CI 0.11–19.9) were not significantly associated with mortality. Among the prognostic factors of breast cancer associated with time to surgery, smoking and AJCC stage were statistically significant. Smoking patients had a statistically higher risk of death than non-smoking patients (HR = 12.05, 95% CI 2.37–61.3). Patients diagnosed with stage 3 breast cancer were at greater risk of death than the reference group (HR = 24.19, 95% CI 1.83–320.07). Patients with unknown histological differentiation also had a higher risk of death (HR = 1.66, 95% CI 0.28–10.10). We observed no significant effects of age at diagnosis, alcohol consumption, asymptomaticity before surgery, presence of ductal carcinoma, chemotherapy, hormone therapy, radiotherapy, or mammogram screening.

**Table 2:** Multivariate analysis of prognostic factors in breast cancer

Characteristic	Model 1				Model 2			
	n	Death (n)	Multivariate model HR (95% CI)^a^		n	Death (n)	Multivariate model HR (95% CI)^a^	
Time to surgery (weeks)								
0–2	88	4	(reference)		88	4	(reference)	
2–4	14	3	1.31	(0.21–8.22)	114	3	1.33	(0.16–11.31)
4–8	444	1	1.47	(0.11–19.95)	44	1	1.46	(0.10–21.72)
> 8	20	5	6.84	(1.06–44.25)	16	4	6.13	(0.75–50.40)
Age at diagnosis (years)								
> 50	109	5	0.41	(0.08–2.00)	108	4	0.19	(0.03–1.21)
≤ 50	157	8	(reference)		154	8	(reference)	
Alcohol consumption								
Yes	202	12	0.17	(0.01–1.98)	63	1	0.64	(0.06–6.65)
No	64	1	(reference)		199	11	(reference)	
Smoker								
Yes	25	4	12.05	(2.37–61.37)	25	4	15.55	(2.69–89.96)
No	241	9	(reference)		237	8	(reference)	
Asymptomaticity before surgery								
Yes	126	1	0.20	(0.02–1.94)	126	1	0.24	(0.02–2.71)
No	140	12	(reference)		136	11	(reference)	
AJCC stage								
I	101	2	(reference)		100	2	(reference)	
II	132	4	6.58	(0.75–58.00)	131	4	18.45	(1.43–238.25)
III	33	7	24.19	(1.83–320.07)	31	6	45.92	(2.02–1,041.96)
Histological differentiation								
Well/Moderate	77	3	(reference)		77	3	(reference)	
Poor	97	5	0.42	(0.05–3.40)	96	4	0.10	(0.01–1.18)
Unknown	92	5	1.66	(0.28–10.10)	89	5	0.87	(0.29–12.18)
Ductal carcinoma								
Yes	236	10	0.87	(0.15–5.00)	232	9	1.16	(0.19–7.16)
No	30	3	(reference)		30	3	(reference)	
Chemotherapy								
Yes	104	9	1.04	(0.21–5.22)	100	8	0.99	(0.20–5.05)
No	162	4	(reference)		162	4	(reference)	
Hormone therapy								
Yes	174	9	1.22	(0.23–6.37)	172	8	0.06	(0.01–0.48)
No	92	4	(reference)		90	4	(reference)	
Radiotherapy								
Yes	209	4	0.28	(0.05–1.50)	208	3	2.10	(0.29–15.43)
No	57	9	(reference)		54	9	(reference)	
Mammogram								
Yes	45	5	0.43	(0.08–2.30)	44	5	0.37	(0.05–2.75)
No	221	8	(reference)		218	7	(reference)	

*AJCC* American Joint Committee on Cancer

Model 1: All patients including neoadjuvant patients who underwent surgical treatment; Model 2: Patients who underwent surgical treatment but did not receive neoadjuvant chemotherapy

^a^Model adjusted for treatment interval

For Model 2, we excluded patients who received neoadjuvant chemotherapy from the analysis, and no significant difference was observed for risk of death according to time to surgery between Models 1 and 2. Patients who smoked were at greater risk of death than their non-smoking counterparts (HR = 15.55, 95% CI 2.69–89.96). Patients with stage 2 breast cancer (HR = 18.45, 95% CI 1.43–238.25) and stage 3 breast cancer (HR = 45.92, 95% CI 2.02–1041.96) were at greater risk of death than those diagnosed with stage 1 breast cancer. Patients who received hormone therapy had a significantly lower risk of death compared to those who did not (HR = 0.06, 95% CI 0.01–0.48). We found no significant effect of age at diagnosis, alcohol consumption, asymptomaticity before surgery, histological differentiation, presence of ductal carcinoma, chemotherapy, radiotherapy, or mammogram screening (Table 2).

## Discussion

The present study presents estimates of the effect of time to surgery on breast cancer prognosis. When all breast cancer patients, including those who received neoadjuvant chemotherapy, were included in the analysis, patients whose surgery was delayed for more than 8 weeks had a greater risk of death compared to those who underwent surgery within 2 weeks of a confirmed diagnosis. We also found that survival improved for patients who received immediate surgical treatment. Smoking, AJCC stage, and hormone therapy significantly influenced survival as prognostic factors. Our results further indicate that the effect of time to surgery on cancer prognosis was the same in the groups with and without neoadjuvant chemotherapy.

Numerous studies on oncological treatment and prognosis have demonstrated that a delay in curative treatment is associated with unfavorable outcomes, including heightened anxiety in patients [15] and an elevated risk of death [23]. By contrast, expedited time to initial surgical treatment has various survival benefits for cancer patients, especially for those with early stage disease [9]. Studies have shown that delayed time from diagnosis to first surgery also affects the time to the next round of surgical treatment. The interval between surgery and the first treatment and that between neoadjuvant chemotherapy and the next surgery both affect survival [19, 24]. The time between the onset of symptoms and the first hospital visit was also reported to have a significant effect on survival [25]. A recent systematic review on the association between mortality and delayed cancer treatment further highlighted that on average, an 8-week delay in breast cancer surgery increased the risk of death by 17%, with longer delays being more increasingly detrimental [20, 26]. These previous findings are consistent with the results of our study that a longer interval between diagnosis and surgery negatively affects breast cancer survival. Neoadjuvant chemotherapy does not improve overall survival, as demonstrated by the National Surgical Adjuvant Breast and Bowel Project (NSABP) B18 trial [27, 28]. Studies have shown that even early stage patients receiving only neoadjuvant chemotherapy had to undergo surgery within 6 weeks [21]. Moreover, the time taken following completion of neoadjuvant chemotherapy to surgery affects survival in early stage patients [19]. Although the present study included patients with AJCC stage 3 stage cancer, we suggest that surgery should be performed on early stage patients within 8 weeks based on the previous research findings.

The potential prognostic role of smoking in breast cancer has been widely discussed. Although studies have presented conflicting findings regarding the direct effect of smoking on breast cancer survival [29], possibly due to patient heterogeneity, there is a consensus among researchers that smokers diagnosed with breast cancer are at higher risk of mortality than nonsmokers [29–32]. Additionally, smoking affects the incidence and overall prognosis of breast cancer in women [30, 31] and is associated with breast cancer recurrence [31]. Patients who quit smoking following breast cancer diagnosis have a 9% lower risk of death than smokers [32]. This finding suggests that the prognosis depends on whether the patient continues to smoke post-diagnosis and that smoking cessation programs are essential for breast cancer patients.

AJCC stage was another factor in this study strongly associated with breast cancer prognosis in cases of delayed surgery. We found that patients with advanced AJCC stage had worse prognoses. Breast cancer patients who exhibit severe disease progression are often advised to undergo neoadjuvant chemotherapy rather than immediate reconstruction to avoid surgical complications [17]. However, among all patients in this study whose surgery was delayed for more than 8 weeks, including patients undergoing neoadjuvant chemotherapy, stage 3 patients had poorer survival than those stage 1 patients. These findings corroborate the finding that overall survival was lower in stage 2 patients than stage 1 patients with increasing delay in surgery [3]. Clinicians should be cognizant of their patients’ AJCC stage when developing treatment plans and ensure that they perform surgery promptly to optimize survival outcomes.

In this study, hormone therapy was associated with a lower risk of death in breast cancer patients not undergoing neoadjuvant chemotherapy. The type of hormone therapy that patient receive depends on the presence of hormone receptors in the patient; thus, treatment methods vary [33]. Tamoxifen is the most common treatment offered to patients with estrogen receptor-positive breast cancer [34]. The duration of these treatments affects prognosis, including overall survival and the likelihood of progression to metastatic contralateral breast cancer [34]. While we did not observe a significant effect of hormone therapy on the prognosis in the patients, including those who received neoadjuvant chemotherapy, an earlier study reported a positive association with breast cancer prognosis in patients receiving neoadjuvant chemotherapy [35]. There are other relevant studies in the literature pertaining to the prediction of response to hormone therapy [36], associations between breast cancer recurrence and the use of hormone therapy and receptor status [37], and survival effects of hormone therapy [38, 39]. These findings collectively suggest that hormone therapy can be beneficial but discontinuation thereof may result in poor prognosis of breast cancer [40]. Continuous and prompt treatment and surgery while avoiding preventable delays are crucial for effective breast cancer care.

Our study is not without limitations. While patients who received neoadjuvant chemotherapy were included in Model 1 along with the other breast cancer patients, it was not possible to focus exclusively on these patients in a separate analysis due to limited data. We were also unable to examine the effect of hormone receptor status as a prognostic factor despite its recognized impact on treatment and survival in breast cancer. Other prognostic factors were also omitted from this study, which underscores the need for additional analyses to improve the understanding of the influence of time to surgery on breast cancer prognosis. Finally, the small sample size may have reduced statistical power. Therefore, future studies are warranted to replicate the study and more clearly elucidate the findings using a larger sample and broader cohorts of patients.

## Conclusions

Our research has demonstrated that the time from diagnosis to surgery significantly influences breast cancer prognosis, with a longer delay in surgery being associated with a higher risk of death. This study emphasizes the impact of time to surgery on breast cancer prognosis highlighting the importance of timely surgical treatment.

## Data Availability

The datasets used and/or analysed during the current study are available from the corresponding author on reasonable request.

## Abbreviations

AJCC: American Joint Committee on Cancer
EMR: Electronic Medical Records
KUH: Konyang University Hospital
KCCR: Korea Central Cancer Registry
NSABP: National Surgical Adjuvant Breast and Bowel Project
